# Review on the Oncology Practice in the Midst of COVID-19 Crisis: The Challenges and Solutions

**DOI:** 10.31557/APJCP.2021.22.1.19

**Published:** 2021-01

**Authors:** Pedram Fadavi, Mohammad Houshyari, Amir Shahram Yousefi Kashi, Alireza Mosavi Jarrahi, Farnaz Roshanmehr, Mohammad Ali Broomand, Saleh Sandoughdaran, Farzad Taghizadeh-Hesary

**Affiliations:** 1 *Department of Radiation Oncology, Iran University of Medical Sciences, Tehran, Iran. *; 2 *Department of Clinical Oncology, Shohada-e Tajrish Hospital, Shahid Beheshti University of Medical Sciences, Tehran, Iran. *; 3 *Cancer Research Center, Faculty of Medicine, Shahid Beheshti University of Medical Sciences, Tehran, Iran. *; 4 *Faculty of Science and Engineering, Waseda University, Tokyo, Japan. *; 5 *Kagawa Nutrition University, Saitama, Japan. *; 6 *Department of Clinical Oncology, Shahid Sadoughi University of Medical Sciences, Yazd, Iran. *; 7 *Shohada Tajrish Hospital, Shahid Beheshti University of Medical Sciences, Tehran, Iran. *; 8 *Department of Clinical Oncology, Shahid Beheshti University of Medical Sciences, Tehran, Iran. *

**Keywords:** Cancer care, - COVID-19, pandemic

## Abstract

As of late 2019, the outbreak of novel coronavirus disease (COVID-19) –that started in China– has rapidly afflicted all over the world. The COVID-19 pandemic has challenged health-care facilities to provide optimal care. In this context, cancer care requires special attention because of its peculiar status by including patients who are commonly immunocompromised and treatments that are often highly toxic. In this review article, we have classified the main impacts of the COVID-19 pandemic on oncology practices –followed by their solutions– into ten categories, including impacts on (1) health care providers, (2) medical equipment, (3) access to medications, (4) treatment approaches, (5) patients’ referral, (6) patients’ accommodation, (7) patients’ psychological health, (8) cancer research, (9) tumor board meetings, and (10) economic income of cancer centers. The effective identification and management of all these challenges will improve the standards of cancer care over the viral pandemic and can be a practical paradigm for possible future crises.

## Introduction

Since December 2019, a novel coronavirus designated as severe acute respiratory syndrome coronavirus 2 (SARS-CoV-2) emerged in the city of Wuhan, China, and spread rapidly over the boundaries to cause the pandemic of viral pneumonia (so-called novel coronavirus disease or COVID-19) in just three months (Burki, 2020; Dewi et al., 2020). The ultra-rapid expansion of the coronavirus has prompted the authorities to find quick and efficient solutions for the ensuing issues. One of these issues is the negative impacts of the viral pandemic on the common oncology practice, which has prompted the community oncologists to search for approaches to maintain their practices while improving the quality of care for patients (Kumar et al., 2020; Poonia et al., 2020; Yadav et al., 2020b). In this regard, experts from all over the world have presented international and national guidelines to cope with the crisis and to provide adjusted care (Ameri et al., 2020a; Ameri et al., 2020b; Dabkara et al., 2020; Miltiadou et al., 2020; Mousavi et al., 2020; Prajoko and Supit, 2020; Rakhsha et al., 2020a; Rakhsha et al., 2020b; Shankar, 2020; Shankar et al., 2020; Siavashpour et al., 2020; Singh et al., 2020; Taghizadeh-Hesary and Akbari, 2020). Oncology centers can follow the guidelines to develop the standards of practices. However, the clinical decision in the midst of the COVID-19 era is not the only case, and it might be overshadowed by several other factors. This review provides a comprehensive look over the main impacts of COVID-19 on oncology practices and how to tackle them. 

*Impacts and Resolutions*

Early studies reported that chemotherapy within the past four weeks slightly increases the mortality of COVID-19 (Yang et al., 2020), however, further cohort studies showed no significant effect of recent anticancer therapies (including radiotherapy, cytotoxic chemotherapy, endocrine therapy, or immunotherapy) on the COVID-19 mortality (Kuderer et al., 2020; Lee et al., 2020; Vuagnat et al., 2020). In addition, a recent meta-analysis clearly shows that a four-week delay in cancer treatment is associated with a 10% increased risk of death across all common cancers (Hanna et al., 2020b). Going by these findings, it would be ideally expected not to modify the treatment of cancer over the pandemic; however, the following shortcomings would make this objective far beyond our wildest dreams. 

*1.1. Impact on Health care Providers*

The COVID-19 pandemic has resulted in a huge burden on the health system for its easy transmission and high mortality rate (Okell et al., 2020). Epidemiological studies have revealed that the rate of hospitalization, intensive care unit (ICU) admission, and case fatality rate of patients with COVID-19 are approximately 20%, 9%, and 3.3%, respectively (Almasi-Hashiani et al., 2020; Basu, 2020). The health infrastructure is further influenced by the fact that health care providers (HCPs) constitute up to 11% of COVID-19 cases (Jeremias et al., 2020). In addition, re-deployment of staff towards critical care to manage patients with COVID-19 further influences cancer care. A weakened health system – with priorities shifting to manage the viral pandemic – will have short- and long-term impacts on the preventive, diagnostic, and therapeutic management of cancer. 

*1.2. Resolutions*

HCPs are crucial to any health-care system; therefore, policymakers have to enact strategies to support exposed and infected HCPs over the pandemic. These strategies are classified into risk stratification, preventive measures, active clinical monitoring, easy access to diagnostic tests, and relevant decision on removal from and return to work. In this context, the HCPs of oncology wards are considered high-risk and they need effective personal protective equipment (PPE), screening with symptoms as well as SARS-CoV-2 diagnostic tests, application of standard isolation duration, and effective treatments (Bielicki et al., 2020). 

*2.1. Impact on Medical Equipment*

During the pandemic, health-care facilities are confronted with engagement of medical equipment with huge number of patients with probable or confirmed COVID-19 that may negatively affect cancer care. This issue may involve all types of medical equipment, including diagnostic (e.g., medical imaging machines and medical laboratory equipment), therapeutic (e.g., linear accelerators), and life supportive (e.g., medical ventilators) equipment. Medical centers may also encounter shortage of hospital beds that subsequently will impair inpatient cancer care. In addition, the necessity of cleaning and disinfecting of medical equipment further limits their application for coronavirus-non-infected patients (e.g., cancer patients) to specified time limits. 

*2.2. Resolutions*

Health-care facilities can manage this issue by allocating a proportion of the number or the time of their equipment to the patients with COVID-19. The latter would be readily available by assigning coronavirus-non-infected patients to one end of the day, preferably in the morning after last night cleaning and disinfection of the devices and environment (Rezaei et al., 2020). 

*3.1. Impact on Access to Medications*

Drug shortage is an ongoing challenge for cancer centers in a time of crisis. This issue is mainly due to budget allocation to provide the necessary medication for the management of COVID-19 and its complications. The cancer drug shortage may have major negative impacts on patients’ survival (e.g., chemotherapeutics in the adjuvant setting or colony-stimulating factors in case of fever and neutropenia) or quality of life (e.g., narcotics or antiemetics). In an analysis of cancer centers from 54 countries, 9.83% (of total 356 centers) have confirmed drug shortage during the pandemic (Ventola, 2011). Even in a nonepidemic situation, intravenous cancer drugs topped the list of medication shortages (Barlas, 2011). This might be due to the fact that these medications are more vulnerable to Good Manufacturing Practice (GMP) violations issued by the world health organization (WHO-GMP), U.S. Food and Drug Administration (CGMP), or European Union (EU-GMP) (Ventola, 2011). In addition, increased procurement time, scarcity of alternative providers, supply chain interruptions, and delays in delivery times may further impair access to biological and chemical oncology drugs over pandemic.

*3.2. Resolutions*

The management of drug shortages is complicated over the viral pandemic, especially in the oncology domain. The specific situation of oncology –that necessitates the provision of medications without interruption– requires advanced strategies to deal with the issue. In 2018, the American Society of Health-System Pharmacists (ASHP) provided recommendations for managing drug shortages, including (1) formation of a working group, (2) validation of drug shortage, (3) formation of resource allocation committee, (4) patient prioritization, (5) finding and approving alternative medications or therapeutic equivalents, and (6) addressing ethical consideration for alternative choices (Fox and McLaughlin, 2018). By following this guideline, health-care facilities can mitigate the impact of the viral pandemic on the provision of necessary medications and reduce its adverse effects on patient care. 

*4.1. Impact on Treatment Approaches*

As the COVID-19 pandemic worsens, health care delivery has been tremendously modified in all disciplines, including oncology. The immunosuppressive nature of cancer treatments has put the oncologists in the great pressure to make the difficult decision of the best care over the pandemic, which would not compromise the patients’ quality and quantity of life (Haghighat and Dehghani, 2020; Mishra et al., 2020). 

*4.2. Resolutions*

In the current unprecedented situation, the approaches settled by health-care facilities to manage the challenge fall into three categories. First, for survivorship and surveillance visits, oncologists can remotely visit patients using telephone calls or video applications. This practice may also include patients who are receiving cancer treatments with low-risk toxicity profile. Second, in cases who the survival benefit of cancer therapy is minimal, oncologists may choose the best supportive care instead. In the remaining patients that treatment is more beneficial, oncologists can apply myriads of modifications to the treatment to minimize hospital visits and decrease patients’ exposure in the medical facilities. Examples would include, hypofractionated radiotherapy instead of conventional schedules, chemotherapy regimen with fewer cycles, less toxic chemotherapy regimens, and using oral rather than parenteral medications (Haresh et al., 2020; Rakhsha et al., 2020a; Schrag et al., 2020). Although several studies declared that cancer therapy in several specified cases (e.g. many prostate cancers, many neuroendocrine tumors, some central nervous system tumors, some thyroid cancers, and some lymphomas) can safely be postponed for 8 to 12 weeks (Schrag et al., 2020), the recent meta-analyses demonstrated opposite findings for several other malignancies (including head and neck, bladder, breast, lung, colorectal, and cervical cancers). In the latter group, minimizing delays to treatment is essential to improve cancer survival rates (Hanna et al., 2020b). Overall, oncologists need to assess the pros and cons of cancer treatment over the pandemic on a case-by-case basis. 

*5.1. Impact on Patients Referral*

As COVID-19 emerged, patients’ referral to the oncology centers has been halted for several reasons. First, clinicians may opt to defer the nonemergency surgeries (including elective cancer surgeries) due to low hospital/ICU bed capacity, shortage of ventilators, limited supplies of PPE, and risk of nosocomial coronavirus transmission. On the other hand, opponents believe that the deferring of elective cancer surgeries has adverse impacts on patients’ survival (Hanna et al., 2020b). Second, the pandemic may also suspend the screening programs in an overwhelmed health care system, which is associated with a higher rate of cases with advanced stage at diagnosis and worse prognosis in the future (Maringe et al., 2020). Third, patients’ anxieties –concerning risk of infection with coronavirus– can also impact their health-seeking behavior and impedes their referral for cancer screening or following up a recent clinical symptom. Last but not least, the lockdown situation can further impair the patients’ referral by preventing inter-city travels of patients seeking the best cancer care. 

*5.2. Resolutions*

To counteract this issue, health-care facilities can take several measures. First, providing rapid and accurate COVID-19 testing before surgery and diagnostic services (e.g., screening colonoscopy, mammography, etc.), assigning dedicated operating rooms and screening clinics with dedicated staff, and guaranteeing sufficient availability of PPE for staff and patients to decrease the concerns of viral transmission and sustain the surgical and screening care over the pandemic. Second, To mitigate the additional cancer deaths resulting from delayed diagnosis, authorities must try to improve public awareness on the risks of not seeking timely medical care for their warning symptoms suggesting malignancy (Maringe et al., 2020). Third, local health-care authorities need to provide the resources to meet the existing and projected needs of cancer care over the not so short COVID-19 era. 

*6.1. Impact on patients Accommodation*

The accommodation is a necessity for patients who traveling away from their homes to get the best cancer care. Over the viral pandemic, however, this may be compromised by assigning the existing premises to patients with COVID-19 and making patients with cancer face extra emotional and financial burden. 

*6.2. Resolutions*

To solve this problem, policymakers in concert with charity organizations can help health-care facilities to provide specified free places for the accommodation of patients with cancer during their treatment. In addition to the lodging of patients, testing of SARS-CoV-2 prior to accommodation, allocation of private rooms to cancer patients and their caregivers, using appropriate air conditioning systems (Rezaei et al., 2020), and paying special attention to the guest services (e.g. food and beverage, cleaning, etc.) and interactions with staff and other patients are crucial to decrease viral transmission in the premises. 

*7.1. Impact on Patients’ Psychological Health*

The emotional threats of the COVID-19 pandemic have been affecting people all over the world, particularly patients with cancer. In this context, cancer patients are vulnerable to a variety of psychological threats, such as anxiety, depression, and cognitive disorders that would adversely affect their quality of life and can induce the onset of symptoms (Adams et al., 2018; Swainston et al., 2020). This critical issue might be initiated by quarantine and lockdown situations –that increase their social isolation and loneliness– and disruption of cancer treatment services (as explained in section 1-6) resulting in exacerbation of patients’ emotional distress. 

*7.2. Resolutions*

The adverse effects of the COVID-19 outbreak on cancer patients’ emotional and cognitive health should not be ignored. With the aforementioned concerns in mind, experts propose to (1) maintain interpersonal relationships –that has powerful therapeutic effects on patients’ emotional health during the hospitalization, quarantine, or lockdown situations– through several ways (e.g. by phone or video calls with family members, psychological support with health care providers, etc.) (Garutti et al., 2020; Passchier et al., 2020); (2) apply telemedicine to decrease patients’ concern over the quarantine (or lockdown) situation regarding the treatment toxicities, follow up evaluation, along with others; (3) give reassurance to the patients to relieve their negative thoughts (e.g. misplaced worries over negative effect of cancer treatment on COVID-19 mortality) and to encourage treatment compliance (Garutti et al., 2020; Lee et al., 2020); (4) reduce the period of quarantine and lockdown situations by authorities; and (5) follow the solutions explained in sections 1-6 to decrease the probability and duration of treatment disruption and patients’ anxiety. 

*8.1. Impact on Cancer Research*

Any of the restrictions explained in the preceding sections can negatively affect the cancer clinical researches by impeding patients’ accrual to the clinical trials and obtaining biospecimens (Singh and Prasad, 2020). In response to the viral pandemic, many cancer centers have transformed their field of interest to COVID-19-directed studies. This change is also evident for a large percentage of oncology journals by allocating numerous special issues to the COVID-19 subject and putting the review process of the articles about COVID-19 in priority.

*8.2. Resolutions*

In response to the COVID-19 crisis, research and therapeutic communities around the world are united to provide and share guidelines. To that end, the European Society of Medical Oncology (ESMO) has provided free online accessible protocols on how to maintain cancer care over the viral pandemic. In addition, oncologists have published their own experiences on the management of site-specific malignancies to provide a guide for their colleagues in the pandemic situation (Dietz et al., 2020; Guckenberger et al., 2020; Hanna et al., 2020a; Siavashpour et al., 2020; Thomson et al., 2020; Zaorsky et al., 2020). Also, several oncologists have opted to apply their experiences in the treatment of cancer toward the management of COVID-19; for example, use of low-dose whole-lung radiotherapy in the treatment of COVID-19 pneumonia (Ameri et al., 2020a; Ameri et al., 2020b; Hess et al., 2020). 

In the midst of the COVID-19 pandemic, a number of main cancer research centers have decided to continue their trials considering specific preparations. For instance, (1) in Dana-Farber Cancer Institute in Boston, researchers have tried to decrease the patient-staff exposures by using phone or video calls to follow up their patients remotely; (2) the researchers of the University of Arizona Cancer Center have applied similar approach and have limited their trials to those that are potentially life-prolonging. They also prescreen the patients via telephone calls for COVID-19 symptoms and recent exposure history before visits; (3) in the Perlmutter Cancer Center in New York, researchers have stratified the trials in order that putting phase 2 or 3 clinical trials in priority and halting early-phase ones. In addition, they have restricted the trials to the outpatient setting; and (4) the researchers of the Yale Cancer Center in New Haven, have followed a similar strategy by continuing ongoing trials and halting new ones (Ribas and Leng, 2020). 

*9.1. Impact on Cancer Multidisciplinary Team Meetings*

The multidisciplinary team meetings (MDTM) in oncology are held to provide the best evidence-based care for patients in terms of diagnosis, treatment, and survivorship. As such, the literature demonstrates the survival improvement with the MDTM approach to cancer care (Kung and Tsai, 2014; Dharmarajan et al., 2020). The COVID-19 pandemic, however, has complicated to hold in-person MDTM due to the possibility of viral transmission (Yadav et al., 2020a).

*9.2. Resolutions*

Prior experience with virtual multidisciplinary team conferences (MDTC) has assisted the clinicians to overcome the shortcoming of in-person MDTM over the COVID-19 pandemic. The utilization of virtual MDTC is associated with decreased delays in cancer care (Stevenson et al., 2013; Salami et al., 2015), more convenient referral coordination (Billingsley et al., 2002), decreased travel burden (Stevenson et al., 2013), and the possibility to hold more frequent sessions (Salami et al., 2015).

*10.1. Economic Impacts*

The complications addressed in the previous sections may cause either decrease in the total income of cancer centers (due to reduced number of treated patients) or an increase in expenditures (e.g. by providing PPE for staff and patients) that eventually result in a negative financial balance of cancer centers during the viral pandemic (Yoshino et al., 2020). This issue may lead to a vicious cycle by disrupting the issues that are essential for cancer centers to continue serving; for instance, the periodic and preventive maintenance and ad-hoc repair of the radiotherapy equipment, the provision of medications, along with others. 

*10.2. Resolutions*

The governments and charity organizations must provide support to help medical institutions (including cancer centers) tide over the harsh economic condition over the COVID-19 era. 

In conclusion, during the COVID-19 era, the health system has to look beyond controlling the pandemic to effectively respond to all the health requirements of the general population, including cancer care. Toward this end, the current review article provides a step in that direction by highlighting ten main issues of cancer care during the viral pandemic. To solve these challenges and maintain cancer patients’ quality and quality of life, the health communities require concerted cooperation of other organizations, including government and charities. On a positive note, the solutions –given in the preceding sections– to improve the standards of cancer care over the pandemic can be a practical paradigm for possible future crises.
